# Failure to Launch: The Endocrine Disruptor Screening Program at the U.S. Environmental Protection Agency

**DOI:** 10.3389/ftox.2022.908439

**Published:** 2022-05-30

**Authors:** Maricel V. Maffini, Laura N. Vandenberg

**Affiliations:** ^1^ Independent Consultant, Frederick, MD, United States; ^2^ University of Massachusetts—Amherst, School of Public Health and Health Sciences, Amherst, MA, United States

**Keywords:** endocrine disrupting chemical, validation, test guideline, cumulative effect, xenoestrogen, FIFRA, Delaney clause, Food Quality Protection Act

## Abstract

It has been 25 years since the U.S. Congress passed the Food Quality Protection Act of 1996, an amendment to the Food Drug and Cosmetic Act, which mandated that the US Environmental Protection Agency (EPA) test all pesticide chemicals used in food for endocrine disruption. Soon after the law passed, EPA established the Endocrine Disruptor Screening and Testing Advisory Committee (EDSTAC) to provide recommendations to the agency on how its Endocrine Disruptor Screening Program (EDSP) should work. Among them, the committee recommended that EDSP screening should 1) evaluate both human and ecological effects; 2) test for disruption of the estrogen, androgen, and thyroid systems; 3) evaluate pesticide and non-pesticide chemicals; and 4) implement a tiered approach. EPA adopted the recommendations and the EDSP was created in 1998. To date, the EPA has yet to fully implement the law; in other words, it has failed to test all pesticide chemicals for endocrine disruption. Of the small number that have been tested, not a single pesticide chemical has been determined to be an endocrine disruptor, and no regulatory actions have been taken. Here, we review the missed opportunities EPA had to make the EDSP a functional and effective program aimed at protecting human health and the environment. Two reports by the EPA’s Office of Inspector General from 2011 to 2021 provide the framework for our discussion.

## Introduction

In the 1960s, the publication of Rachel Carson’s *Silent spring* brought to the attention of the American public concerns that had been raised by researchers and environmental advocates alike, that environmental pollution was significantly and adversely impacting the health of wildlife and humans (Carson, 1987). Yet, it took several more decades for researchers to understand that many of these environmental pollutants were adversely affecting health by altering the actions of hormones including androgens, estrogens and thyroid hormones. In fact, from the 1960s through the early 1990s, numerous examples illustrated that pharmaceutical and environmental agents that alter hormone actions could detrimentally affect the health of individuals and populations (Sharpe and Skakkebaek, 1993; Newbold and Mclachlan, 1996; Sumpter, 1998; Kogevinas, 2001; Tan and Zoeller, 2007).

From the late 1980s to the mid-1990s, the science of what it is now known as endocrine disruption grew steadily with contributions from numerous scientific disciplines including conservation biology, cancer biology, endocrinology, and toxicology, among others (Wingspread Conference et al., 1992; Colborn et al., 1993; Schug et al., 2016). It took a series of mutually reinforcing events, as described by Dr. Sheldon Krimsky in his book *Hormonal Chaos,* to support a scientific movement to investigate the impact of endocrine disrupting chemicals (EDCs) on the health of human populations (Krimsky, 2003). These events included a National Academy of Science report examining pesticides in children’s diets (National Research Council, 1993), and attention from the lay public after release of the BBC documentary “Assault on the male”, and publication of the book *Our Stolen Future* (Colborn et al., 1995). With the public’s attention captured, and concern raised by the increasing number of studies identifying EDCs and their harmful effects, Congress encouraged regulatory action by the US Environmental Protection Agency (EPA) through the passage of several bills.

In this Perspective, we will review the steps that led to the development of a screening program to be implemented by the EPA to ensure the protection of the public from hormonally active pesticides. Unfortunately, as the EPA Inspector General concluded in its two evaluations of the program, the lack of strategic management plan and misplaced expectations about testing assays led to long delays that would prevent the EPA’s program from achieving this important goal. Ultimately, fewer than 75 pesticides would be screened and, to our knowledge, none has been regulated as an EDC.

## Congress Acknowledges the Problem of Endocrine-Active Pesticides

In 1993, a bill was introduced in the House of Representatives which aimed to speed up the re-registration of pesticides that had not been properly evaluated previously (Krimsky, 2003). During the congressional hearings held prior to its passage, emphasis was placed on human and wildlife health effects of estrogen-mimicking chemicals by both the committee co-chairs and scientists that provided testimony, including Drs. Theo Colborn, Ana Soto and Louis Guillette.

**FIGURE 1:**
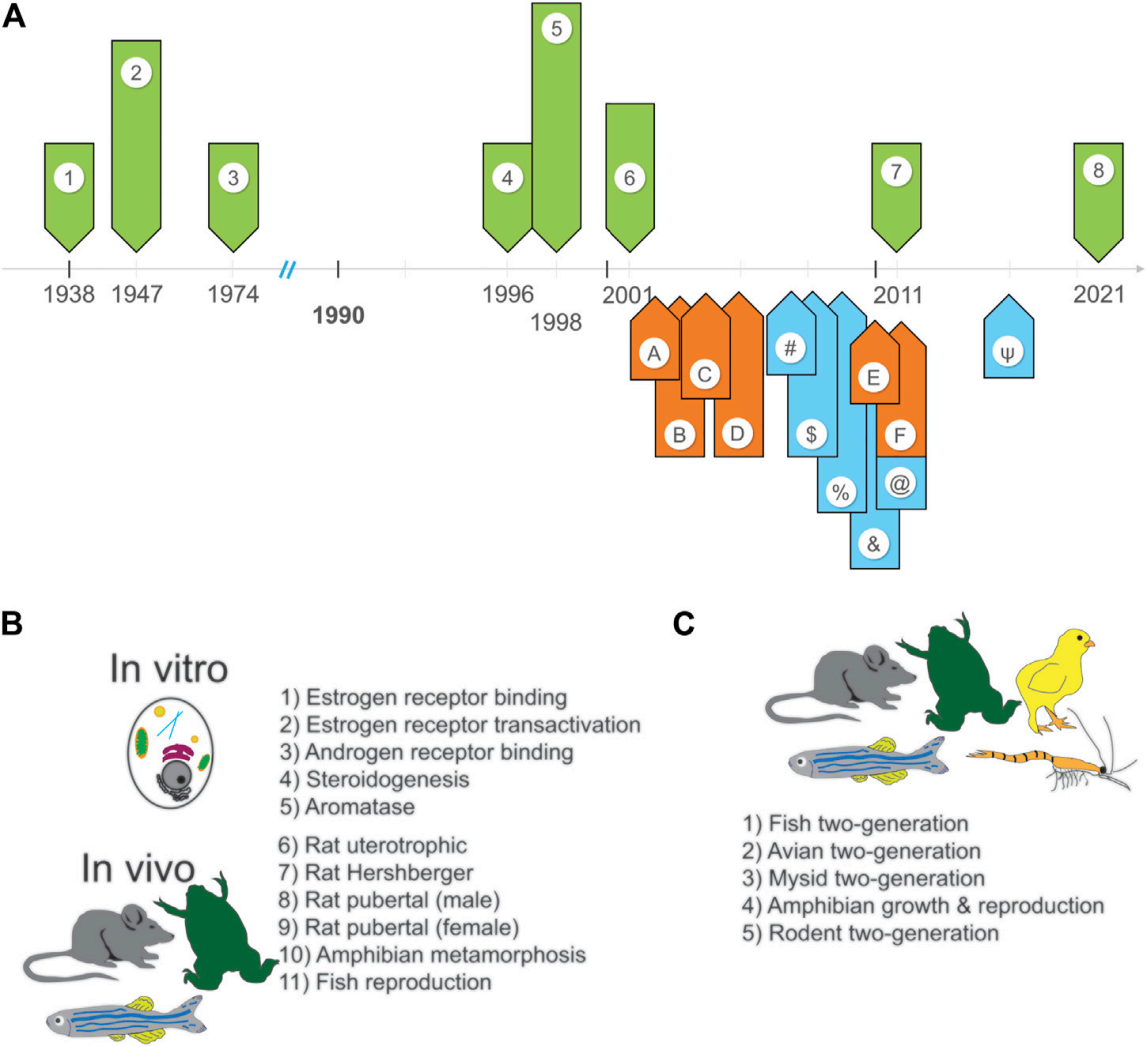
Overview of the ongoing implementation of the EDSP. **(A)** A timeline of the events leading up to the development, creation, and review of the EDSP (green arrows), the promised or proposed timeline for completion of specific tasks (orange arrows), and the achievement of specific tasks (blue arrows). The code for these events is as follows: Events: 1—FDCA passes (1938); 2—FIFRA passes (1947); 3—Safe Drinking Water Act passes (1974); 4—FQPA passes, EDSTAC committee is assembled (1996); 5—EDSTAC final report is published (1998); 6—Lawsuit filed by Natural Resources Defense Council is settled (2001); 7) IG’s first report is published (2011); 8) IG’s second report is published (2021). Promises: A—to publish initial list of chemicals to evaluate (2002); B—to validate all Tier one assays except frog metamorphosis, begin testing (2003); C—validate the mammalian 2-generation test and start Tier two testing (2004); D—validate all other Tier two tests (2005); E—publish a second list of chemicals to evaluate (2010); F—evaluate Tier one data on 67 chemicals from first list (2011). Achievements: #—EPA releases draft list of chemicals to evaluate (2007); $—EPA completes validation of Tier one assays and has them peer-reviewed (2008); %—EPA releases guidelines for Tier one assays and publishes final list of chemicals (2009); &—EPA produces a second list of chemicals for evaluation (2010); @—EPA validates non-mammalian Tier two tests (2011); ψ—EPA finalizes Tier two tests, evaluates Tier one data for 52 chemicals from the first list (2015). **(B)** Tier one assays include *in vitro* and *in vivo* screens. **(C)** Tier two tests utilize numerous species.

It took three more years and multiple negotiations until the Food Quality Protection Act of 1996 (FQPA) was signed into law (Food Quality Protection Act, 1996). The FQPA amended two existing laws: the Federal Insecticide, Fungicide, and Rodenticide Act of 1947 (FIFRA) and the Food, Drug and Cosmetic Act of 1938 (FDCA). See Figure 1A for a more complete timeline. The new law required the EPA to develop a screening program specifically to determine if chemicals used in pesticides had estrogenic properties.

Prior to the passage of FQPA, multiple bills had been introduced that included provisions related to EDCs. Alongside legislative initiatives, the science of endocrine disruption and the understanding of the risks posed by chemicals with the ability to interfere with hormonal systems, including pesticides, continued to grow. Yet, some scientists, conservative think tanks, and concerned industry and agricultural groups argued that EDCs pose no threat to human or environmental health (Bailey, 1996; Cato Institute, 1996). Since then, the evidence of EDCs threatening human and environmental health has only grown stronger (Gore et al., 2015).

A major reason the pesticide industry and agribusiness supported the FQPA was because it replaced the Delaney clause, a hazard-based approach to evaluate pesticide residues in food, with a risk-based approach. The Delaney clause is part of the FDCA and it states that “no additive shall be deemed to be safe if it is found to induce cancer when ingested by man or animal, or if it is found, after tests which are appropriate for the evaluation of the safety of food additives, to induce cancer in man or animal. . .” (21U.S.C. § 348(c) (3) (A)). Although this standard was eliminated for pesticide residues in food, the Delaney clause remains a requirement for all other food chemicals (Neltner et al., 2011).

## The FQPA at a Glance

The FQPA was designed to standardize EPA’s management of pesticides (Bergeson and Hutton, 2004) and included new regulatory responsibilities for EPA to protect human health. The main amendments to the FDCA included:

1. Health-based safety standards for pesticide residues in food;
2. Special provisions to protect infants and children;
3. Limitations to the consideration of pesticide ‘benefits’;
4. Review of existing pesticide tolerances (i.e., maximum permissible pesticide residue on treated food) as well as uniformity of tolerances; and
5. Screening and testing for endocrine disruption.

Regarding screening and testing of chemicals, the FQPA gives EPA the power to require a company that registers a pesticide with the agency (e.g., the manufacturer or importer), to test for endocrine disruption and impose penalties if the company fails to comply.

The main changes to FIFRA included:

1. Requiring pesticide reregistration every 15 years. In other words, EPA routinely re-evaluates the risk of the pesticide considering new hazard and exposure data;
2. The development of procedures to expedite review of safer pesticides; and
3. Require review and registration of antimicrobial pesticides.

Screening for EDCs was front and center at the time and language from the FQPA was also added to the Safe Drinking Water Act, the law that protects drinking water (Safe Drinking Water Act, 1996).

## The EPA’s First Task: Develop a Screening Program for EDCs

The FQPA gave EPA the mandate to develop and implement a screening program to determine whether some chemicals may negatively affect human health by disrupting the endocrine system. To meet the new requirement, the EPA assembled a federal advisory committee, the Endocrine Disruptor Screening and Testing Advisory Committee (EDSTAC) in 1996 (EDSTAC, 1998). Comprised of research scientists from academia, government and public health groups and advocates from the chemical industry, EDSTAC recommended the creation of an endocrine disruptor screening program (EDSP). They proposed that the EDSP should focus on chemicals that bind to the estrogen (E), androgen (A) and thyroid hormone (T) receptors, while also acknowledging that endocrine science was developing rapidly and the screening program would need to include additional hormone pathways in the future.

In their 1998 final report, EDSTAC acknowledged several important issues that were relevant to EDCs and other environmental chemicals (EDSTAC, 1998; Baltz, 1999). First, there were more than 80,000 chemicals in use; although 25,000 would be unlikely to interact with hormone receptors due to their size and/or physiochemical properties, tens of thousands would need to be evaluated. EDSTAC recommended that these chemicals be prioritized based on exposure data, physiochemical properties, or hazard/toxicity data. Second, EDSTAC recommended that the EDSP utilize a two-tiered approach:

• Tier one would use a screening approach with both *in vitro* and *in vivo* assays, including high-throughput approaches, to identify chemicals with the potential to interact with EAT receptors or chemicals that alter steroidogenesis (Figure 1B).
• Tier two would consist of tests to evaluate endocrine-mediated adverse outcomes (Figure 1C).

Finally, EDSTAC recommended that the EDSP be used not only for the evaluation of chemicals, but also to determine if chemical *mixtures* have endocrine disrupting properties and suggested that six kinds of mixtures be prioritized: breast milk contaminants, phytoestrogens found in soy infant formulas, mixtures commonly found at hazardous waste sites, pesticide and fertilizer mixtures, disinfection byproducts, and contaminants of gasoline.

## The EDSP Is Grounded

Following EDSTAC’s suggestions, EPA began developing the EDSP in the late 1990s with a two-tiered design as described above (Gray, 1998; Schmidt, 1999). EDSTAC had noted that implementation of the EDSP would be challenging in part because of the large Universe of chemicals that required testing, and thus proposed a prioritization scheme (EDSTAC, 1998). To achieve its goals, EPA asked the FIFRA scientific advisory board to create a subcommittee to evaluate EDSTAC’s recommendations (EPA OIG, 2011). In 1999, the FIFRA advisory board recommended that the EPA start by reviewing data for 50–100 pesticides using at least the EDSP Tier one assays (EPA OIG, 2011). This would not happen for more than a decade.

The FQPA required EPA to use validated assays, but the assays included in the EDSP had not been validated at the time the law passed. Assay validation establishes its reliability (i.e., the reproducibility within and between laboratories over time) and relevance (i.e., the degree that a test is meaningful and useful for a specific purpose). In 1999, EPA promised that validation of nine assays for the EDSP Tier one would be completed within a 2-year period and proposed a five step validation process (Schmidt, 1999). As discussed in more detail below, the EPA would fail to meet this deadline by many years. In 2005, international guidance was provided for how assays like those used in the EDSP would be validated (OECD, 2005) including the testing of coded reference chemicals by multiple participating laboratories.

The FQPA required EPA to implement the EDSP by August 1999. This was a very ambitious timeline considering the magnitude of the task and it soon became clear that the agency was taking longer to complete it than was originally prescribed by Congress. EPA’s work was closely scrutinized due to its importance to protect human health, so in August 1999, the Natural Resources Defense Council and other public health advocacy watchdogs sued EPA (NRDC vs. EPA, 1999) for failing to meet the statutory deadline to implement EDSP.

As part of the lawsuit settlement agreement in 2001 (EPA OIG, 2011), EPA committed to a new set of deadlines including:

• December 2002: publication of the first list of chemicals for screening;
• December 2003: validation of Tier one assays (except the frog thyroid assay);
• December 2003: requiring testing for certain Tier one screens;
• December 2004: requiring testing for certain Tier two tests;
• December 2004: validation of Tier two mammalian two-generation assay; and
• December 2005: validation of other Tier two assays.

Although some results were achieved, none of the deadlines were met (EPA OIG, 2011). For instance, Tier one assays were validated in 2008 (5 years behind schedule), and the Tier two mammalian two-generation assay has yet to be validated in 2022 (now 18 years late).

## The EPA Fails to Fully Launch the EDSP

The Office of Inspector General (OIG, 2018) is an independent unit within US government agencies. The office’s goals include promoting efficiency and effectiveness of government agencies and detection of fraud or abuse via periodic evaluation of programs. As such, the EPA’s Inspector General (IG) evaluated the EDSP program and published its first report in 2011 (EPA OIG, 2011). The IG sought to determine whether EPA “has planned and conducted the requisite research and testing to evaluate and regulate” EDCs. The report’s conclusions were not encouraging:

• *“EDSP has made little progress in identifying [EDCs]. While we acknowledge that EDSP encountered difficulties and delays, its lack of progress is also due to EPA’s lack of management control over the program.*”
• *“EDSP will not be able to establish an effective screening and testing program without establishing program control and accountability. As a result, achieving the goal of protecting human health and the environment from [EDCs] will continue to be delayed.”*

The IG provided six specific technical and managerial recommendations to the leadership of the EPA’s Office of Chemical Safety and Pollution Prevention (OCSPP) including establishing the scope of chemicals to include in the program, developing methods to prioritize the chemicals for screening and testing, and finalizing criteria for evaluation of Tier one screening and Tier two testing data received. The report also recommended EPA develop a management plan for EDSP, outcome and output performance measures and annual reviews of progress. Table 1 includes the IG’s recommendations and summarizes EPA’s responses. In short, more than 10 years after it began to implement FQPA, EPA agreed to develop a comprehensive management plan and performance measures. However, the IG considered the agency’s responses to the technical recommendations inconclusive.

**TABLE 1:** Synthesis of the EPA’s Inspector General 2011 and 2021 recommendations to improve the implementation of EDSP.

2011 IG recommendations	EPA Response to Recommendations	Timeline for Completion
1- Define and identify the universe of chemicals for screening and testing to establish the scope of the program	The Agency believes that the scope of the current EDSP is clearly defined by the law. We have already identified the universe of chemicals for screening: all pesticide chemicals and drinking water contaminants	September 2011: Work plan
	EPA intends to use a science-based prioritization process to identify additional chemicals for EDSP screening. EPA will develop a work plan focused on integrating computational toxicology to EDSP to prioritize additional chemicals	June 2012: Management plan
2- Develop and publish a standardized methodology for objectively prioritizing the universe of chemicals for screening and testing, including elements recommended by the federal advisory committees such as use of effects and exposure data	Given the ongoing, scientific research in this area, flexibility will be a key feature of any prioritization methodology so that future developments and alternative approaches can be incorporated as appropriate	September 2011: Work plan
	We anticipate that an initial prioritized list of chemicals could be developed in the near-term using tools such as ToxCast and Quantitative Structure Activity Relationship (QSAR) models in combination with other data	June 2012: Management plan
3- Finalize specific criteria for evaluating the Tier 1 screening data received and establish specific criteria for evaluating the Tier 2/hazard assessment testing data received	The Agency is currently evaluating public comments on the draft criteria for evaluating Tier 1 screening data	September 2011: Finalize criteria
	Agency and the broader scientific community have a long history of conducting hazard and risk assessments of the type envisioned in Tier 2 of the EDSP. However, the Agency plans to develop Standard Evaluation Procedures (SEPs) specific to the individual Tier 2 tests. The Agency cannot develop these SEPs until validation of the Tier 2 tests is completed	December 2012: Completing SEP for each Tier 2 tests
4- Develop short-term, intermediate, and long-term outcome performance measures, and additional output performance measures, with appropriate targets and timeframes, to measure the progress and results of the program	Short-term outcomes could consist of making weight-of-evidence determinations to decide whether a chemical will move on to EDSP Tier 2 testing	June 2012: Release comprehensive management plan including these measures
	Intermediate outcomes could consist of the hazard assessments that will result from Tier 2	
	Long-term outcomes could include a characterization of the regulatory actions that result from EDSP screening and testing, the impact of such actions on human health and the environment and other metrics	
5- Develop and publish a comprehensive management plan for EDSP, including estimates of EDSP’s budget requirements, priorities, goals, and key activities covering at least a 5-year period	The management plan will cover at least 5 years into the future of the EDSP and will include the continued issuance of test orders, the development of a consolidated information infrastructure for the EDSP, and other aspects of the program	June 2012
	It will address budget requirements for the EDSP and performance management, including performance measures and annual reviews	
6- Annually review the EDSP program results, progress toward milestones, and achievement of performance measures, including explanations for any missed milestones or targets	The Agency reports annually on the EDSP’s performance measures. This reporting includes progress toward annual targets with explanations for any that are missed or exceeded	June 2012
	The Agency will continue this review process and will consider additional options for annual program reviews as we develop the comprehensive management plan for the EDSP.	
1- Issue Tier 1 test orders for each List 2 chemical or publish an explanation for public comment on why Tier 1 data are no longer needed to characterize a List 2 chemical’s endocrine-disruption activity	*Proposed Corrective Action 1a*: OCSPP, with input from the Office of Research and Development and the Office of Water, will publish for comment a List 2 Action Plan, which may include a combination of test orders, explanations as to why test orders are not needed, or a reprioritization of the order of EDSP evaluations	30 September 2024 for Action 1a
	*Proposed Corrective Action 1b*: Following notice and comment as described in Corrective Action 1a, OCSPP will initiate the process to issue test orders for List 2 substances, as appropriate	30 September 2025 for Action 1b
2- Determine whether the EPA should incorporate the Endocrine Disruptor Screening Program Tier 1 tests (or approved new approach methodologies) into the pesticide registration process as mandatory data requirements under 40 C.F.R. § 158 for all pesticide use patterns	*Proposed Corrective Action 2*: OCSPP will make a determination on the inclusion of the EDSP Tier 1 tests into the pesticide registration process as mandatory data requirement under 40 C.F.R. part 158 for all pesticide use patterns	30 September 2024
3- Issue List 1–Tier 2 test orders for the 18 pesticides in which additional Tier 2 testing was recommended or publish an explanation for public comment on why Tier 2 data are no longer needed to characterize the endocrine-disruption activity for each of these 18 pesticides	*Proposed Corrective Action 3a*: OCSPP will make a determination on the need for List 1-Tier 2 data. OCSPP will also provide an explanation, which will be published for public comment, for any of the 18 pesticides for which it is determined that Tier 2 data is no longer needed	31 December 2023 for Corrective Action 3a
	*Proposed Corrective Action 3b*: Following publication and comment as described in Corrective Action 3a, OCSPP will initiate the process to issue any Tier 2 test orders for List 1 determined to be needed	30 September 2024 for Corrective Action 3b
4- Issue for public review and comment both the Environmental Fate and Effects Division’s approach for the reevaluation of List 1–Tier 1 data and the revised List 1–Tier 2 wildlife recommendations	*Proposed Corrective Action 4*: OCSPP will issue for public review and comment any reevaluation of List 1–Tier 1 data and any revisions to the List 1–Tier 2 wildlife recommendations	31 December 2023 for completing and posting for public comment together with Proposed Corrective Action 3a
5- Develop and implement an updated formal strategic planning document, such as the Comprehensive Management Plan	*Proposed Corrective Action 5*: OCSPP, with input from the Office of Research and Development and the Office of Water, will develop an EDSP Strategic Plan. OCSPP expects to update this document on an as needed basis	30 September 2022
6- Develop performance measures, with reasonable time frames, to document progress toward and achievement of milestones or targets. Specifically, the Endocrine Disruptor Screening Program should consider at least one performance measure that tracks progress in testing pesticides for human endocrine disruptor activity	*Proposed Corrective Action 6a*: OCSPP will develop short-term performance measures, such as scientific publications, number/type of accepted new approach methods, and exemptions granted	Short-term performance measures under Proposed Corrective Action 6a will be developed by and tracked beginning 1 October 2022
	*Proposed Corrective Action 6b*: OCSPP will develop longer-term performance measures, including at least one measure to track progress in testing pesticides for human endocrine disruptor activity	Long-term performance measures under Proposed Corrective Action 6b including at least one that tracks progress in the evaluation and testing of pesticides for human endocrine disruptor activity will be developed and tracked by 1 October 2024
7- Conduct annual internal program reviews of the Endocrine Disruptor Screening Program	*Proposed Corrective Action 7*: OCSPP will conduct the first annual internal program review of the EDSP and provide a briefing and report out to the OCSPP Assistant Administrator on EDSP progress, especially as it relates to the Corrective Actions in this Report and progress developing the EDSP Strategic Plan	30 September 2022
8- Complete and publish the Endocrine Disruptor Screening Program’s response(s) to 2015 Federal Register notice comments and its related white paper	*Proposed Corrective Action 8*: OCSPP will complete and publish the response to 2015 Federal Register notice comments and the NAM White Paper	December 2021
9- Establish a procedure for Endocrine Disruptor Screening Program communications and coordination with relevant Agency program offices with testing responsibilities	*Proposed Corrective Action 9*: OCSPP will establish a procedure for communications and coordination with relevant Agency program offices with EDSP testing responsibilities	30 September 2021
10- To increase external communication and transparency, update the Endocrine Disruptor Screening Program website, including the program timeline, and publish any relevant program documents	*Proposed Corrective Action 10*: The EDSP will update the EDSP website to post the response to the 2015 Federal Register notice comments and the NAM White Paper on the Endocrine Disruptor Screening Program website	Corrections to the EDSP website, including hyperlinks to documents and other webpages, have already begun. The NAM White Paper and associated documents will be published on the Endocrine Disruptor Screening Program website by 30 December 2021
	Continuing updates, for example on the OCSPP reorganization, will also be done as needed to increase external communication and transparency	

Shaded rows: Management recommendations. OCSPP: Office Chemical Safety and Pollution Prevention. NAM: New alternative methods. EDSP: endocrine disruption screening program.

## After a Second IG Review, EDSP Shows Very Limited Results

In 2021, 25 years after FQPA passed and 10 years after its first report, the EPA’s IG published its second evaluation of EDSP (EPA OIG, 2021). The report states that more than 1,300 chemicals had been determined to be high priority for evaluation because of their use as pesticides, yet only a small percentage had been considered for screening or testing:

• In 2011, EPA issued testing orders for only 52 chemicals to be evaluated with Tier one assays;
• In 2015, EPA determined that Tier two testing was needed for 18 of the 52 chemicals evaluated in the first round, but failed to issue testing orders;
• In 2013, EPA published a second list of 109 chemicals it recommended to evaluate with Tier one assays, but never issued testing orders for these chemicals. Tier two tests were also not ordered.

The conclusion from the 2021 IG’s report was straightforward: use of the EDSP has stalled (EPA OIG, 2021). Furthermore, the IG noted additional concerns, including the appearance of bias in the EPA’s evaluation of data from the first 52 chemicals. The report found that the EPA had changed its approaches to evaluate data from Tier one assays after receiving data on the first 52 chemicals. Finally, the IG report included a shocking conclusion that “some EPA staff indicated that they were instructed to function as if the EDSP was eliminated from the EPA’s budget.” It is worth noting that in fiscal year 2021, EDSP was allocated US $7.5 million with only four full-time staff members.

## Is It Too Late to Get the EDSP off the Ground?

Looking back over the last 25 years, there were numerous opportunities to protect people, especially children, and the environment from EDCs. First, Congress crafted a strong law that considered numerous aspects of endocrine disruption science including non-linear dose responses and the absence of thresholds. Second, EPA was mandated to develop a strong and sustainable program to prioritize and evaluate pesticides used on food and other chemicals to determine which have endocrine disrupting properties. Although the EDSP would eventually focus on chemicals that act via the EAT receptors, the FQPA acknowledged that endocrine science was more complex, and testing programs would need to adapt as new mechanisms of action were observed. In spite of the strong law and support from Congress, the EPA would fail to meet numerous deadlines, and has still not completed its evaluation of the first set of chemicals that were selected for testing using the EDSP in 2007.

Today, EDSP still does not have a strategic plan with priorities and guidance; several of the tests to be included in the EDSP have not been validated, and the approaches that have been used to examine some data raised questions about bias, leading the IG to conclude that “the EPA risks losing credibility with the public that its decisions are impartial” (EPA OIG, 2021).

In its response to the 2021 evaluation, current leadership at EPA laid out what appears to be a new turn in the tortuous path of EDSP implementation. It stated that “in the last decade, EPA focused its efforts on developing new approach methods (NAMs) because of “the extensive resources (time, cost, and use of laboratory animals) required to develop and evaluate the Tier 1-List one data.” The EPA claims that computational and *in vitro* testing are faster and more efficient and provide “more human-relevant data”. EPA plans to develop case studies to evaluate how to use NAM data in combination with Tier one and two studies submitted for pesticide registration and other relevant data. However, EPA is already behind its 2021 deadline to announce the acceptance and use of these NAMs (see Table 1).

Furthermore, the first IG report (EPA OIG, 2011) expressed concern at the EPA’s plan to replace Tier one assays with non-validated screening tests (i.e., those included in the EPA’s ToxCast program), noting “[o]nce the Agency is able to validate the use of ToxCast tests for screening chemicals, it will be appropriate to include it in the EDSP management plan. Until that time, the Agency should include how it will use its existing proven (validated) test procedures to screen chemicals in the EDSP comprehensive management plan” (EPA OIG, 2011). That same criticism is relevant to the NAMs, which are not yet validated.

Despite the recommendations made following a 2011 IG report and corrective actions that were promised by EPA leadership, the agency has failed to implement remediations to make the program work. Worse still, staff were told to ignore the legal requirement to create a screening program to identify EDCs. Based on EPA’s response to the 2021 IG report, it is hard to imagine whether EDSP will ever take off. Collectively, these failures continue to put the public at risk, and question whether a modern screening program will ever be successful in identifying and regulating EDCs in the United States.

## References

[B1] Bailey R. (1996). Hormones and Humbug. Washington Post: Washington, D.C.

[B2] Baltz D. (1999). Endocrine Disruption Comes into Regulatory Focus. New Solut. 9, 29–35. 10.2190/3ym8-34by-br0b-9ctw 17208914

[B3] Bergeson L. L. Hutton C. N. (2004). The Food Quality Protection Act-Implementation and Legal Challenges. Envtl. L. Rep. News Analysis 34, 10733.

[B4] Carson R. (1987). Silent Spring. 25th Anniversary Edition. New York: Houghton Mifflin.

[B5] Cato Institute (1996). Let Science Judge the Sperm Crisis. Washington, D.C: Cato Institute. Available at: https://www.cato.org/commentary/let-science-judge-sperm-crisis .

[B7] Colborn T. Dumanoski D. Myers J. P. (1995). Our Stolen Future. New York: Penguin Books.

[B8] Colborn T. Vom Saal F. S. Soto A. M. (1993). Developmental Effects of Endocrine-Disrupting Chemicals in Wildlife and Humans. Environ. Health Perspect. 101, 378–384. 10.1289/ehp.93101378 8080506 PMC1519860

[B9] EDSTAC (1998). Endocrine Disruptor Screening and Testing Advisory Committee (EDSTAC) Final Report. Washington, D.C: US Environmental Protection Agency.

[B10] EPA OIG (2021). EPA's Endocrine Disruptor Screening Program Has Made Limited Progress in Assessing Pesticides. Washington, D.C: US EPA Office of the Inspector General. Available at: https://www.epa.gov/office-inspector-general/report-epas-endocrine-disruptor-screening-program-has-made-limited .

[B11] EPA OIG (2011). EPA's Endocrine Disruptor Screening Program Should Establish Management Controls to Ensure More Timely Results. Washington, D.C: US EPA Office of the Inspector General. Available at: https://www.epa.gov/office-inspector-general/report-epas-endocrine-disruptor-screening-program-should-establish .

[B12] Food Quality Protection Act. 1996. Food Quality Protection Act of 1996. Public Law 104–170.

[B13] Gore A. C. Chappell V. A. Fenton S. E. Flaws J. A. Nadal A. Prins G. S. (2015). EDC-2: The Endocrine Society's Second Scientific Statement on Endocrine-Disrupting Chemicals. Endocr. Rev. 36, E1–E150. 10.1210/er.2015-1010 26544531 PMC4702494

[B14] Gray L. E. Jr. (1998). Tiered Screening and Testing Strategy for Xenoestrogens and Antiandrogens. Toxicol. Lett. 102-103, 677–680. 10.1016/s0378-4274(98)00287-2 10022334

[B15] Kogevinas M. (2001). Human Health Effects of Dioxins: Cancer, Reproductive and Endocrine System Effects. Apmis 109, S223–S232. 10.1111/j.1600-0463.2001.tb05771.x 11392380

[B16] Krimsky S. (2003). Hormonal Chaos: The Scientific and Social Origins of the Environmental Endocrine Hypothesis. Baltimore, MD: Johns Hopkins University Press. 10.1038/73073 10700215

[B17] National Research Council (1993). Pesticides in the Diets of Infants and Children. Washington (DC): National Academies Press. 25144038

[B18] Neltner T. G. Kulkarni N. R. Alger H. M. Maffini M. V. Bongard E. D. Fortin N. D. (2011). Navigating the U.S. Food Additive Regulatory Program. Compr. Rev. Food Sci. Food Saf. 10, 342–368. 10.1111/j.1541-4337.2011.00166.x

[B19] Newbold R. R. Mclachlan J. A. (1996). Transplacental Hormonal Carcinogenesis: Diethylstilbestrol as an Example. Prog. Clin. Biol. Res. 394, 131–147. 8778794

[B20] NRDC vs EPA (1999). Northern District of California. San Francisco, CA: US District Court for the Northern District of California. Available at: https://archive.epa.gov/pesticides/regulating/laws/fqpa/web/pdf/nrdcdecree2.pdf .

[B21] OECD (2005). “Guidance Document on the Validation and International Acceptance of New or Updated Test Methods for Hazard Assessment,” in Number 34. OECD Series on Testing and Assessment Number 34 (Paris, France: Organisation for Economic Co-operation and Development).

[B22] Office of the Inspectors General (2018). Offices of the Inspectors General. Washington, D.C: US Government. Available at: https://www2.ed.gov/about/offices/list/oig/misc/authorities-responsibilities1.pdf .

[B23] Safe Drinking Water Act. (1996). Safe Drinking Water Act Amendment of 1996. Public Law 104–182.

[B24] Schmidt C. W. (1999). Answering the Endocrine Test Questions. Environ. Health Perspect. 107, A458–A460. 10.1289/ehp.99107a458 10464085 PMC1566461

[B25] Schug T. T. Johnson A. F. Birnbaum L. S. Colborn T. Guillette L. J. Jr Crews D. P. (2016). Minireview: Endocrine Disruptors: Past Lessons and Future Directions. Mol. Endocrinol. 30, 833–847. 10.1210/me.2016-1096 27477640 PMC4965846

[B26] Sharpe R. M. Skakkebaek N. E. (1993). Are Oestrogens Involved in Falling Sperm Counts and Disorders of the Male Reproductive Tract? Lancet 341, 1392–1396. 10.1016/0140-6736(93)90953-e 8098802

[B27] Sumpter J. P. (1998). Xenoendocrine Disrupters - Environmental Impacts. Toxicol. Lett. 102-103, 337–342. 10.1016/s0378-4274(98)00328-2 10022275

[B28] Tan S. W. Zoeller R. T. (2007). Integrating Basic Research on Thyroid Hormone Action into Screening and Testing Programs for Thyroid Disruptors. Crit. Rev. Toxicol. 37, 5–10. 10.1080/10408440601123396 17364703

[B6] Wingspread Conference (1992). “Wingspread Consensus Statement,” in Chemically Induced Alterations in Sexual and Functional Development: The Human/wildlife Connection. Editors Colborn T. Clement C. (Princeton: Princeton Scientific Publishing).

